# Dirac semimetal phase and switching of band inversion in *X*Mg_2_Bi_2_ (*X* = Ba and Sr)

**DOI:** 10.1038/s41598-021-01333-z

**Published:** 2021-11-09

**Authors:** Daichi Takane, Yuya Kubota, Kosuke Nakayama, Tappei Kawakami, Kunihiko Yamauchi, Seigo Souma, Takemi Kato, Katsuaki Sugawara, Shin-ichiro Ideta, Kiyohisa Tanaka, Miho Kitamura, Koji Horiba, Hiroshi Kumigashira, Tamio Oguchi, Takashi Takahashi, Kouji Segawa, Takafumi Sato

**Affiliations:** 1 Department of Physics, Graduate School of Science, Tohoku University, Sendai, 980-8578 Japan; 2 Precursory Research for Embryonic Science and Technology (PRESTO), Japan Science and Technology Agency (JST), Tokyo, 102-0076 Japan; 3 Center for the Promotion of Interdisciplinary Education and Research, Kyoto University, Kyoto, 606-8501 Japan; 4 Center for Spintronics Research Network, Tohoku University, Sendai, 980-8577 Japan; 5 Advanced Institute for Materials Research (WPI-AIMR), Tohoku University, Sendai, 980-8577 Japan; 6 UVSOR Synchrotron Facility, Institute for Molecular Science, Okazaki, 444-8585 Japan; 7 School of Physical Sciences, The Graduate University for Advanced Studies (SOKENDAI), Okazaki, 444-8585 Japan; 8 Hiroshima Synchrotron Radiation Center, Hiroshima University, Higashi-Hiroshima, 739-0046 Japan; 9 Institute of Materials Structure Science, High Energy Accelerator Research Organization (KEK), Tsukuba, Ibaraki 305-0801 Japan; 10 National Institutes for Quantum and Radiological Science and Technology (QST), Sayo, Hyogo 679-5148 Japan; 11 Institute of Multidisciplinary Research for Advanced Materials (IMRAM), Tohoku University, Sendai, 980-8577 Japan; 12 Center for Spintronics Research Network, Osaka University, Toyonaka, 560-8531 Japan; 13 Institute of Scientific and Industrial Research, Osaka University, Ibaraki, Osaka 567-0047 Japan; 14 Department of Physics, Kyoto Sangyo University, Kyoto, 603-8555 Japan

**Keywords:** Topological insulators, Electronic properties and materials

## Abstract

Topological Dirac semimetals (TDSs) offer an excellent opportunity to realize outstanding physical properties distinct from those of topological insulators. Since TDSs verified so far have their own problems such as high reactivity in the atmosphere and difficulty in controlling topological phases via chemical substitution, it is highly desirable to find a new material platform of TDSs. By angle-resolved photoemission spectroscopy combined with first-principles band-structure calculations, we show that ternary compound BaMg_2_Bi_2_ is a TDS with a simple Dirac-band crossing around the Brillouin-zone center protected by the C_3_ symmetry of crystal. We also found that isostructural SrMg_2_Bi_2_ is an ordinary insulator characterized by the absence of band inversion due to the reduction of spin–orbit coupling. Thus, *X*Mg_2_Bi_2_ (*X* = Sr, Ba, etc.) serves as a useful platform to study the interplay among crystal symmetry, spin–orbit coupling, and topological phase transition around the TDS phase.

## Introduction

Topological Dirac semimetals (TDSs) materialize a new state of quantum matter which hosts exotic quantum phenomena such as high carrier mobility^1,2^, giant linear magnetoresistance^1^, and Fermi-arc-mediated surface transport^3,4^. Such physical properties are governed by the Dirac-cone energy band showing linear dispersions in all the directions in the three-dimensional (3D) momentum (*k*) space (*k*_*x*_, *k*_*y*_, and *k*_*z*_) characterized by the crossing of bulk valence band (VB) and conduction band (CB) at discrete points (Dirac points) in *k* space. Such peculiar electronic structure is formed as a result of the band inversion due to the strong spin–orbit coupling (SOC) and the protection of band degeneracy by the time-reversal and specific crystalline symmetries. The most intriguing aspect of TDS is that it can be switched to other topological states such as quantum spin Hall states, topological insulators (TIs), Weyl semimetals, and topological superconductors^5–12^. For instance, breaking the time-reversal symmetry by an external magnetic field leads to a Weyl semimetal state possessing a pair of spin-split massless Weyl cones in the bulk and novel Fermi arcs at the surface^13,14^. Another way with a potentially high degree of freedom in manipulating the topological state is chemical substitution which may enable, e.g. to control the Dirac-cone dispersion via tuning SOC or to trigger a topological superconducting state with Majorana fermions by carrier doping. Angle-resolved photoemission spectroscopy (ARPES) has played a pivotal role in clarifying TDSs by directly visualizing the bulk Dirac-cone band, as demonstrated in Na_3_Bi, Cd_3_As_2_, and α-Sn/InSb(111)^5,7,15–18^ which host Dirac-cone bands protected by the rotational symmetry. Despite many theoretical predictions for TDS candidates^19–29^, experimental investigations *hitherto* performed on TDSs are mainly focused on these prototypical TDSs, which, however, have inherent problems such as high reactivity in the atmosphere and/or difficulty of chemical substitution of elements in crystals. Thus, it is highly desirable to explore new TDS materials which overcome such difficulties.

**Figure 1:**
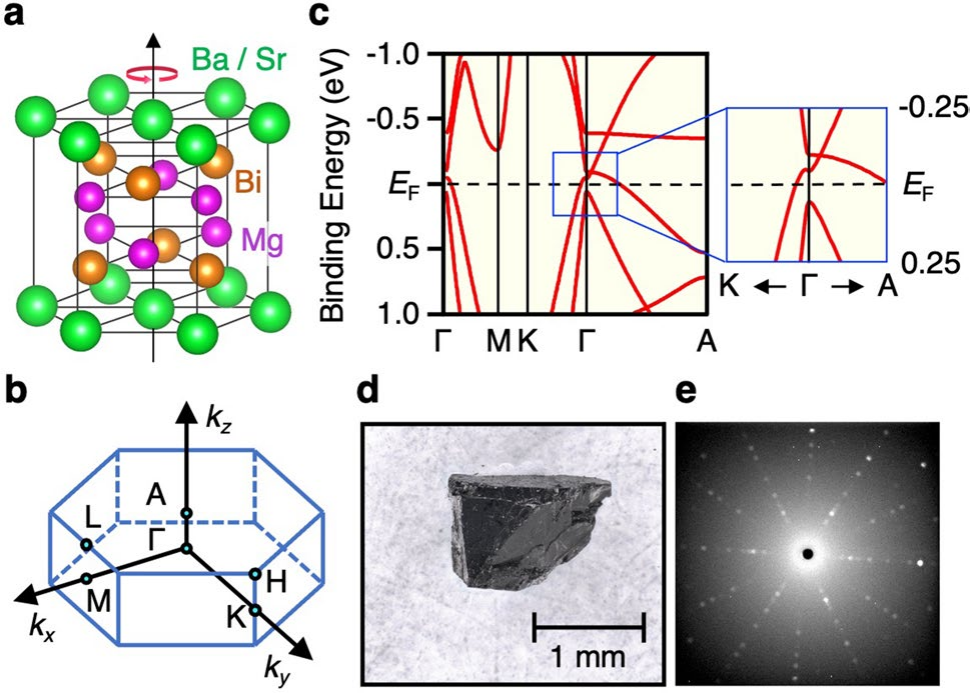
(**a**,**b**) Crystal structure and bulk hexagonal BZ of *X*Mg_2_Bi_2_ (*X* = Sr and Ba), respectively. (**c**) Calculated band structure of BaMg_2_Bi_2_ along high-symmetry lines in bulk BZ obtained from the first-principles band-structure calculations, and its expanded view around the Dirac point (right panel). (**d**) Photograph of BaMg_2_Bi_2_ single crystal. (**e**) X-ray Laue backscattering image of BaMg_2_Bi_2_ obtained for the (0001) surface.

Recently, it was proposed that a ternary compound BaMg_2_Bi_2_ is a 3D TDS^30^ from the automated construction of Wannier functions and high-throughput screening out of non-topological materials. In BaMg_2_Bi_2_ crystal, Ba atoms can be easily replaced with other elements, and its general chemical composition is expressed as *X*Mg_2_Bi_2_ where *X* represents alkaline earth or rare earth metal (Ca, Sr, Ba, Yb, Eu, etc.)^31–35^. *X*Mg_2_Bi_2_ crystalizes in the CaAl_2_Si_2_-type structure (space group of *P*$\overline{3}$m1, No. 164^36^ which has the C_3_ rotational symmetry with respect to the *c*-axis (Fig. 1a; the Brillouin zone (BZ) is shown in Fig. 1b). Our first-principles band-structure calculations including SOC shown in Fig. 1c reveal that BaMg_2_Bi_2_ possesses a Dirac cone close to the Fermi level (*E*_F_) along the ΓA line of bulk BZ (Fig. 1b) due to the bulk-band inversion and the protection by the C_3_ symmetry, consistent with the previous calculation^30^. The predicted simple band structure with no *E*_F_ crossings of other bands with topologically trivial origin makes BaMg_2_Bi_2_ an excellent system to search for exotic properties associated with bulk Dirac fermions. Moreover, the *X*Mg_2_Bi_2_ family has a high potential to realize different types of topological states. For example, controlling the SOC by replacing the *X* element and breaking the crystal symmetry e.g. by applying pressure may trigger the topological phase transition to other phases such as TI and ordinary insulator phases^30,37^. Despite such interesting proposals, the electronic states of *X*Mg_2_Bi_2_ family have been scarcely investigated. It is thus urgently required to clarify its fundamental band structure.

In this work, we report high-resolution angle-resolved photoemission spectroscopy (ARPES) of *X*Mg_2_Bi_2_ (*X* = Sr and Ba) single crystals. By utilizing energy-tunable soft-X-ray (SX) and vacuum ultraviolet (VUV) photons from synchrotron radiation, we experimentally established the electronic structure of BaMg_2_Bi_2_ over the 3D bulk BZ and found an evidence for the existence of bulk Dirac fermions characterized by the band touching of bulk VB and CB. In sharp contrast, we found a bulk-band gap in SrMg_2_Bi_2_ suggestive of its ordinary insulator nature. We discuss the obtained results in relation to the band-structure calculation, SOC, and topological phase transition.

## Results and discussion

### Samples and experimental

*X*Mg_2_Bi_2_ single crystals with typical size of 2 × 2 × 1 mm^3^ (see Fig. 1d) were synthesized by the self-flux method (for details, see “Methods”). ARPES measurements were performed with micro-focused VUV synchrotron light at BL28 in Photon Factory and BL5U in UVSOR, and also with SX photons at BL2 in Photon Factory. Samples were cleaved in situ along the (0001) plane of the hexagonal crystal in an ultrahigh vacuum of 1 × 10^–10^ Torr. Prior to ARPES measurements, the crystal orientation was determined by the X-ray Laue backscattering measurements which signify clear six-fold symmetric diffraction spots consistent with the (0001) cleaved plane, as shown in Fig. 1e.

### Valence-band structure of BaMg_2_Bi_2_

**Figure 2:**
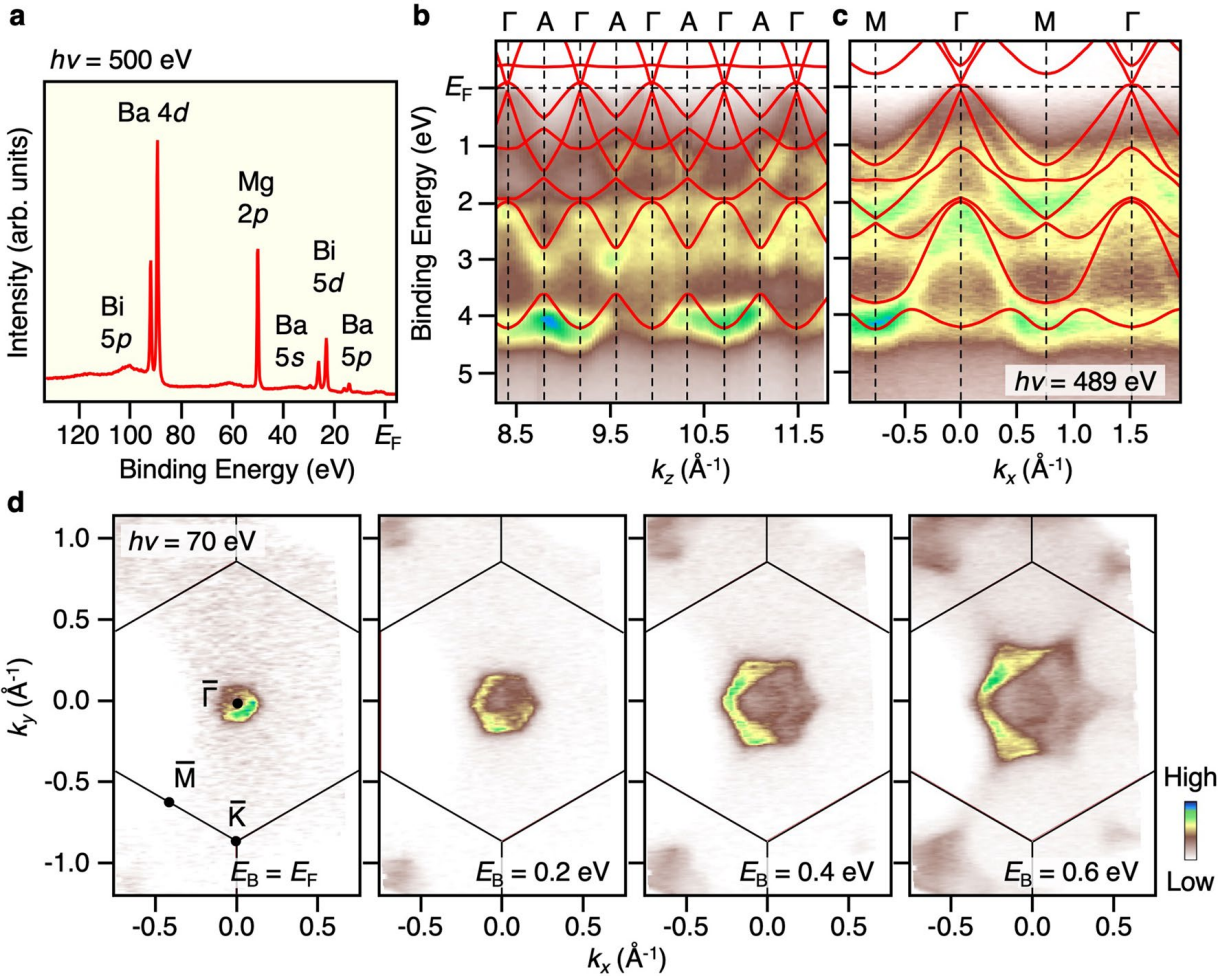
(**a**) Energy distribution curve of BaMg_2_Bi_2_ in a wide binding-energy (*E*_B_) range measured at *hν* = 500 eV. (**b**) ARPES intensity of BaMg_2_Bi_2_ measured at *T* = 40 K along the ΓA cut by varying *hν* from 250 to 522 eV, together with the calculated band structure (red curve). The inner potential was estimated to be *V*_0_ = 15.0 eV from the periodicity of out-of-plane band dispersion. (**c**) ARPES intensity along the $\overline{{\Gamma \text{M}}}$ cut measured at *hν* = 489 eV. Calculated band structure along the ΓM cut is also overlaid. (**d**) Plots of ARPES intensity as a function of two-dimensional wave vector (*k*_*x*_ and *k*_*y*_) at representative *E*_B_ slices (*E*_B_ = 0, 0.2, 0.4, and 0.6 eV) measured at *hν* = 70 eV for BaMg_2_Bi_2_.

We at first discuss the overall electronic structure of BaMg_2_Bi_2_. Figure 2a displays the energy distribution curve in a wide energy range measured at the photon energy (*hν*) of 500 eV. One can recognize several core-level peaks which are assigned to the Ba (5*s*, 5*p*), Mg (2*p*), and Bi (5*p*, 5*d*) orbitals. The sharp spectral feature and the absence of core-level peaks from other elements confirm the clean sample surface. Besides the core levels, one can find a weak feature near *E*_F_ originating from the VB composed mainly of the Bi 6*p* orbitals. To determine the 3D bulk electronic states, we performed ARPES measurements at the normal-emission setup with varying *hν* in the SX region (250–522 eV). To visualize the bulk band in 3D *k* space, it is useful to use bulk-sensitive SX photons, because the increase of photoelectron mean-free path compared to VUV photons reduces the intrinsic uncertainty of the out-of-plane wave vector *k*_*z*_ through the Heisenberg's uncertainty principle and as a result allows the accurate 3D band mapping. Figure 2b shows the obtained VB dispersion along the wave vector perpendicular to the sample surface (*k*_*z*_). One can recognize some energy bands displaying a finite *k*_*z*_ dispersion, e.g., at the binding energies *E*_B_’s of *E*_F_-1.5 eV, 2–3 eV, and 3.7–4.3 eV. The observed band dispersions well follow the periodicity of the bulk BZ; for example, the near-*E*_F_ band appears to have the top and bottom of dispersion at Γ and A points, respectively, consistent with the bulk-band calculations including SOC (red curves). A good matching of periodic oscillation in the band dispersion between the experiment and calculation is seen in the entire *E*_B_ region, showing their bulk-origin nature.

Since the VB has the top of dispersion around the Γ point of bulk BZ, next we carried out ARPES measurements along the in-plane *k*_*x*_ cut crossing the Γ point (ΓM cut), as shown in Fig. 2c. One can recognize several bands whose energy dispersion is reproduced semi-quantitatively by the band calculation along the ΓM cut, suggesting no discernible band-mass renormalization, indicative of a weak electron correlation in BaMg_2_Bi_2_. One can also see two hole bands which rapidly disperse toward *E*_F_ with approaching the Γ point. The outer one appears to cross *E*_F_ and forms a hole pocket centered at the Γ point. To visualize the topmost VB more clearly, we plot in Fig. 2d the ARPES intensity as a function of in-plane wave vector, *k*_*x*_ and *k*_*y*_, at selected *E*_B_ slices (*E*_B_ = *E*_F_, 0.2, 0.4, 0.6 eV) at *k*_*z*_ ~ 0 (in reduced BZ scheme; *k*_*z*_ = 7 × 2π/*c* in extended BZ), measured with VUV photons (*hν* = 70 eV) that achieve higher energy and *k* resolution. At *E*_B_ = *E*_F_, one can clearly see a circular Fermi surface centered at the Γ point. This Fermi surface follows the periodicity of hexagonal BZ (for details, see Supplementary Fig. S1). With increasing *E*_B,_ the circular intensity pattern gradually expands and shows a stronger hexagonal deformation, signifying that the topmost VB forms a hole pocket, consistent with the positive sign of Hall coefficient in our sample (see Supplementary Fig. S2). Taking into account the sizable *k*_*z*_ dispersion of the topmost VB (Fig. 2b), it is suggested that the hole pocket has the 3D character. By assuming an isotropic spherical shape, we have estimated the hole carrier concentration to be 7 × 10^18^ cm^−3^. This value is in rough agreement with the value estimated from the Hall coefficient (2.4 × 10^18^ cm^−3^ at *T* = 50 K; for details, see Supplementary Sect. 2 of Supplementary Information), suggesting that the ARPES data reflect intrinsic bulk properties.

**Figure 3:**
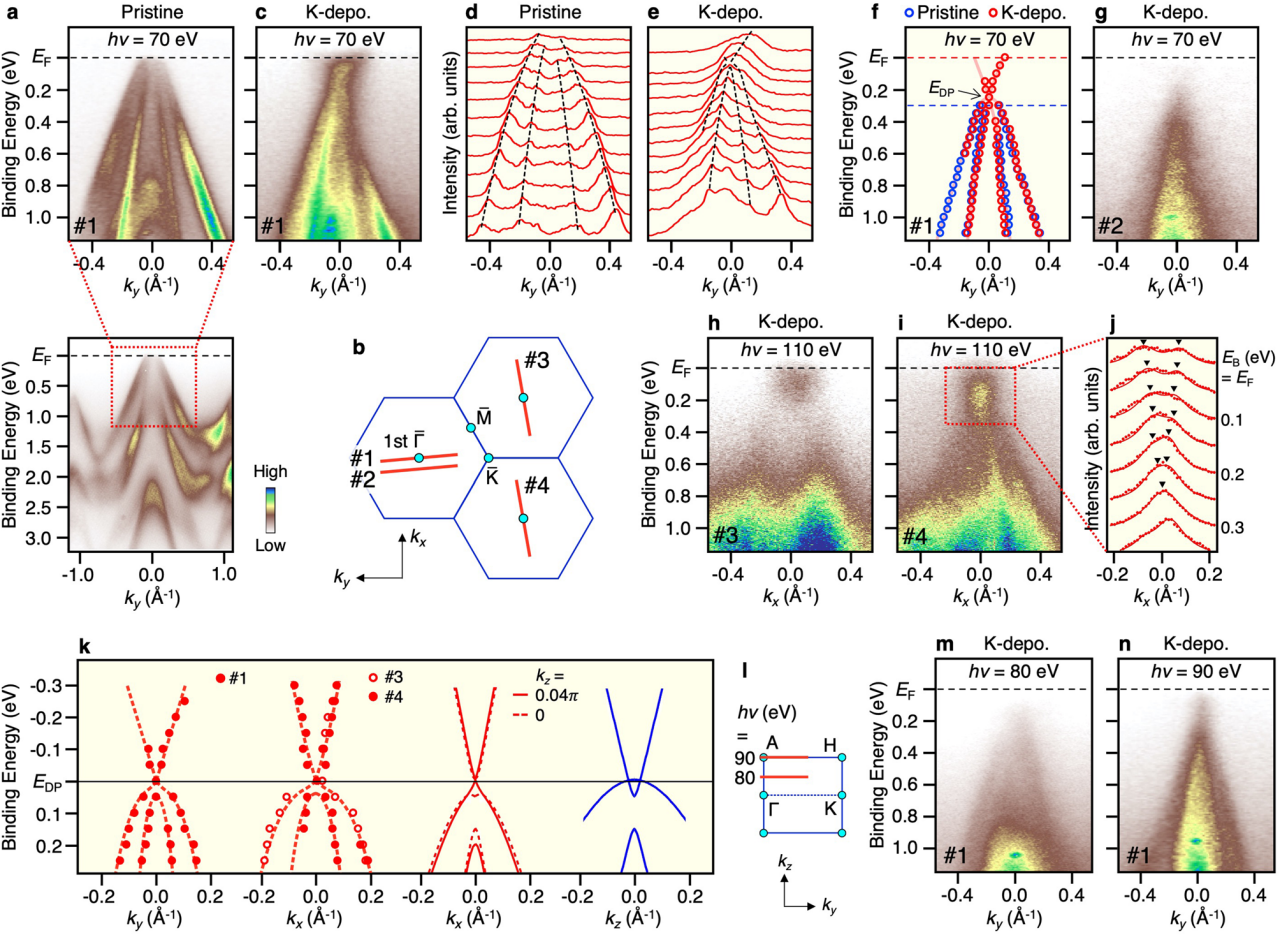
(**a**) VB ARPES intensity (bottom panel) and expanded image in the vicinity of *E*_F_ (top panel) for BaMg_2_Bi_2_ measured nearly along the ΓK cut at *hν* = 70 eV. (**b**) Brillouin zone of BaMg_2_Bi_2_ in the *k*_*x*_–*k*_*y*_ plane together with *k* cuts where the ARPES data shown in (**c**) (cut #1), (**g**) (cut #2), (**h**) (cut #3), and (**i**) (cut #4) were obtained. (**c**) ARPES intensity near *E*_F_ measured along cut #1 after K deposition onto the surface of BaMg_2_Bi_2_. (**d**,**e**) MDCs at several *E*_B_’s (*E*_F_-1.1 eV) corresponding to the ARPES intensity of (**a**) and (**b**), respectively. Dashed curves are a guide to the eyes to trace the band dispersion. (**f**) Experimental band dispersions along cut #1 for pristine and K-deposited BaMg_2_Bi_2_, obtained by tracing the peak position of MDCs in (**d**) and (**e**). Band dispersion for pristine sample is shifted downward by 0.3 eV with respect to that of K-deposited counterpart to align the energy position of the Dirac point. (**g**) ARPES intensity measured along cut #2 for K-deposited BaMg_2_Bi_2_. (**h**,**i)** ARPES intensity measured along cuts #3 and #4, respectively, for K-deposited BaMg_2_Bi_2_. (**j**) MDCs in the area enclosed by red rectangle in (**i**). (**k**) Experimental band dispersion (circles and dashed curves) obtained from the MDC analyses along cuts #1, #3, and #4, compared with the calculated band dispersion along the *k*_*x*_ cut at *k*_*z*_ = 0 (ΓM cut; red dashed curve) and 0.04 Å^−1^ (*k* cut crossing the Dirac point; red solid curve), and with that along the *k*_*z*_ axis (ΓA cut; blue curves). Experimental and calculated band dispersions are aligned in energy with respect to the Dirac point. (**l**) Brillouin zone of BaMg_2_Bi_2_ in the *k*_*y*_–*k*_*z*_ plane together with *k* cuts where ARPES measurements shown in panels (**m**) and (**n**) were performed. (**m**,**n**) ARPES intensity for K-deposited BaMg_2_Bi_2_ measured at *hν* = 80 eV and 90 eV, respectively.

Now that the overall VB dispersion is established, we present the near-*E*_F_ band structure to examine the existence of bulk Dirac fermions. Figure 3a shows the ARPES intensity (bottom panel) and its expanded view in the vicinity of *E*_F_ (top panel), measured almost along the ΓK (*k*_*y*_) cut (cut #1 in Fig. 3b) at *hν* = 70 eV. One can see linearly dispersive inner and outer hole bands, and the latter crosses *E*_F_. Since hole carriers are doped into the crystal due to the slight off-stoichiometry of chemical composition, we were unable to see the bulk CB on the bare surface. To search for a possible band touching of VB and CB predicted in the calculation, we deposited K atoms onto the surface of BaMg_2_Bi_2_ in ultrahigh vacuum. One can clearly see in Fig. 3c that the hole bands in K-deposited BaMg_2_Bi_2_ are shifted downward as a whole by ~ 0.3 eV with respect to those in pristine BaMg_2_Bi_2_ due to the electron doping to the surface. Intriguingly, VB in the negative *k*_*y*_ region appears to almost continuously disperse across *k* = 0 and cross *E*_F_ at positive *k*_*y*_, without losing its intensity at *k*_*y*_ = 0, suggestive of the absence of band gap. Taking into account that the linearly dispersive VB at positive *k*_*y*_ shows a weaker intensity due to the dipole matrix-element effect of photoelectron intensity, together with the expectation that the band dispersion is symmetric with respect to *k*_*y*_ = 0, it is suggested that the VB and CB touch each other at the Γ point and forms the Dirac-cone-like dispersion. To corroborate the band touching, we show in Fig. 3d,e the momentum distribution curves (MDCs) corresponding to the ARPES intensity of Fig. 3a,c, respectively. One can see that, in K-deposited BaMg_2_Bi_2_ (Fig. 3e), the peak for the bulk VB is smoothly connected to that for the bulk CB without obvious discontinuity around the Γ point, supporting the absence of an energy gap. This is also visualized by the experimental band dispersion obtained by tracing the peak position of MDCs shown in Fig. 3f. The band touching only at a single *k* point in the *k*_*x*_-*k*_*y*_ plane (i.e., the $\overline{\Gamma }$ point of the surface Brillouin zone) is confirmed by the ARPES intensity measured along the off-$\overline{\Gamma }$ cut (cut #2 in Fig. 3b) in Fig. 3g that signifies the apparent absence of CB due to an energy separation between the CB and the VB, supporting the cone-shaped band dispersion. To further support this conclusion, we have carried out ARPES measurements with different experimental conditions. Figure 3h,i show the representative ARPES intensity after the K deposition on the surface of BaMg_2_Bi_2_ obtained by another cleaving, measured at *hν* = 110 eV nearly along the ΓM cut (#3 and #4, respectively, in Fig. 3b) thus with different light polarization with respect to the sample surface. While the distribution of ARPES intensity is different from that of Fig. 3c (note that somewhat fuzzy character of the ARPES spectrum along the ΓM cut is attributed to the matrix-element effect of photoelectron intensity), these intensity patterns commonly show that CB is located rightly above the VB without a band-gap opening. As shown in Fig. 3j, the plot of MDCs in the *E*_B_ = 0–0.35 eV region of Fig. 3i reveals two broad peaks at *E*_B_ = *E*_F_ (triangles) whose *k* separation becomes gradually smaller with increasing *E*_B_, and the two peaks eventually merge into a single peak at *E*_B_ = 0.3 eV. This signifies the existence of V-shaped CB. We have experimentally determined the energy position of VB and CB from numerical fittings of MDCs in Fig. 3c,h,i and found that outer hole band appears to touch the CB at the Γ point within our experimental uncertainty, as shown in the two panels on the left side of Fig. 3k. The band dispersion determined by ARPES shows an overall agreement with the calculated band dispersions along the ΓM cut (*k*_*z*_ = 0; dashed curves, slightly away from the Dirac point) and off-ΓM cut (*k*_*z*_ = 0.04 Å^−1^; solid curves, passing through the Dirac point) as shown in the second panel from the right in Fig. 3k. As shown in the right panel of Fig. 3k, the calculated Dirac points exist not exactly at the Γ point but slightly (by 0.04 Å^-1^) away from the Γ point along the ΓA cut. By taking into account the finite *k*_*z*_ broadening (0.1–0.2 Å^−1^, estimated from the photoelectron escape depth) which is wider than the *k* separation between adjacent Dirac points, the present ARPES data would reflect the band dispersion integrated over the *k*_*z*_ region involving two Dirac points. Thus, although it is difficult to resolve each Dirac point due to the inevitable *k*_*z*_ broadening effect, the overall agreement between the experimental and calculated band dispersions is consistent with the existence of Dirac points around the Γ point, and therefore supportive of the TDS nature of BaMg_2_Bi_2_. To support the 3D nature of the band touching, we show in Fig. 3m,n the ARPES intensity measured with *hν* = 80 and 90 eV which correspond to *k*_*z*_ ~ 0.5π (between AH and ΓK cut) and π (AH cut), respectively (Fig. 3l). One can recognize that the CB seen at *hν* = 70 eV (along the ΓK cut; Fig. 3c) is absent for both *hν* = 80 and 90 eV because it moves upward and enters the unoccupied region, suggesting that the band touching occurs in a particular *k*_*z*_ point (*k*_*z*_ ~ 0) in the 3D Brillouin zone, indicative of the overall 3D cone-shaped nature of the bulk band dispersion. This also suggests that the influence of K-deposition-induced electron doping extends into the bulk region, so that the quantized two-dimensional CB, as observed in semiconductor surfaces^38^, is not formed in K-deposited BaMg_2_Bi_2_.

### Switching of band inversion

**Figure 4:**
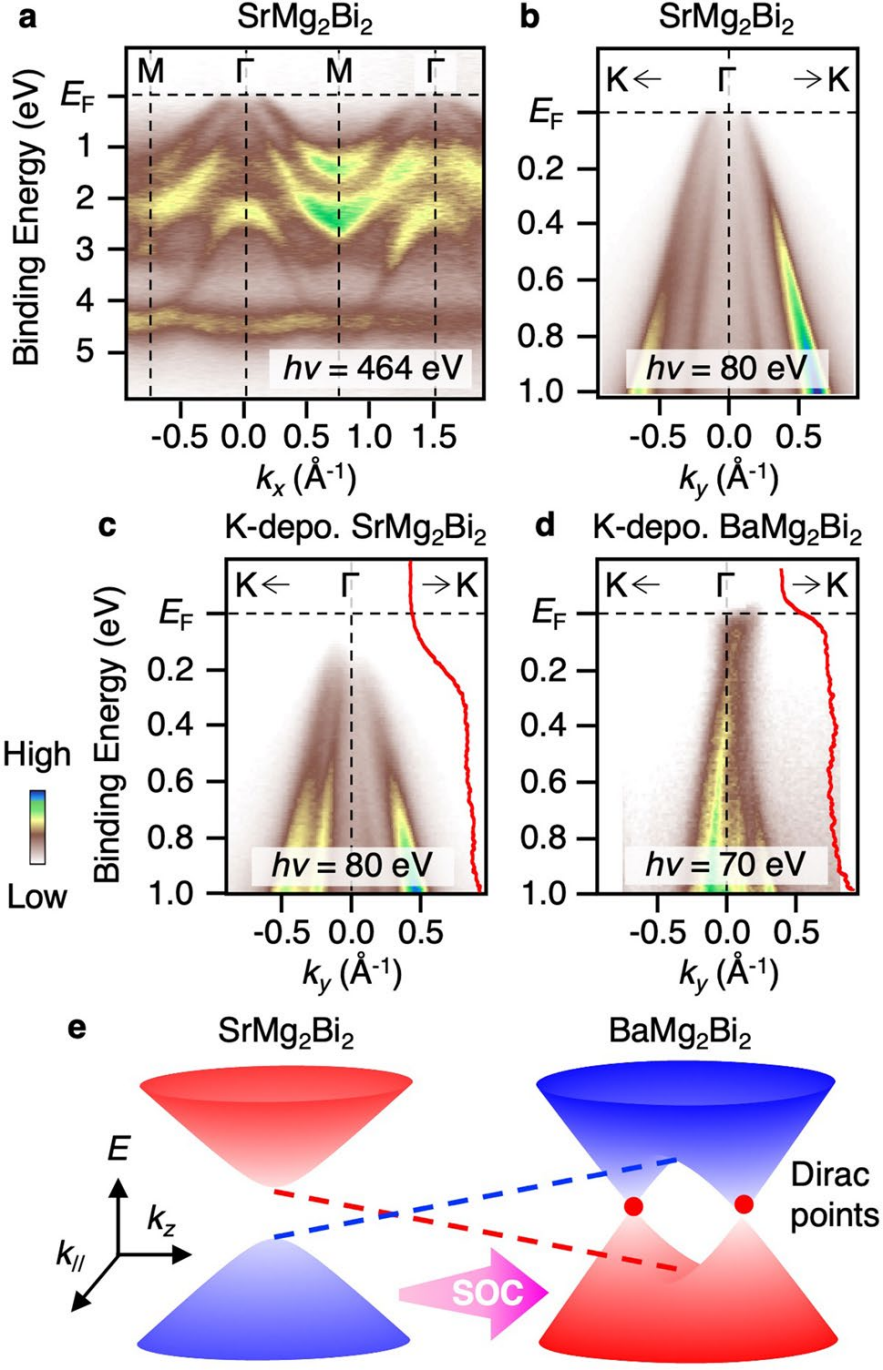
(**a**) VB ARPES intensity of SrMg_2_Bi_2_ measured at *T* = 40 K along the ΓM cut at *hν* = 489 eV. (**b**,**c**) ARPES intensity in the vicinity of *E*_F_ of SrMg_2_Bi_2_ before and after K deposition, respectively, measured almost along the ΓK cut (cut #1 in Fig. 3b) at *hν* = 80 eV. (**d**) ARPES intensity for K-deposited BaMg_2_Bi_2_ (same as Fig. 3c). EDCs at the Γ point are shown in (**c**) and (**d**). (**e**) Schematic band diagram of *X*Mg_2_Bi_2_ suggested from the ARPES results.

Next we address an important question whether or not the electronic structure of isostructural SrMg_2_Bi_2_ is similar to that of BaMg_2_Bi_2_. Figure 4a displays the ARPES intensity along the ΓM cut for SrMg_2_Bi_2_ measured with SX photons (*hν* = 489 eV). One can recognize qualitatively similar VB dispersions to those of BaMg_2_Bi_2_ (Fig. 2c), for example, three prominent hole bands centered at the Γ point and relatively flat band at around 4–4.5 eV, are clearly observed, while there exist some quantitative differences such as a slight downward shift of the flat band in SrMg_2_Bi_2_ relative to BaMg_2_Bi_2_. The topmost hole band crosses *E*_F_ in SrMg_2_Bi_2_, as better visualized in the high-resolution ARPES data in the vicinity of *E*_F_ shown in Fig. 4b measured with VUV photons which probe the Γ point (*hν* = 80 eV). To examine the possible band touching of VB and CB, we deposited K atoms on the surface of SrMg_2_Bi_2_ as in the case of BaMg_2_Bi_2_. As shown in Fig. 4c, although the hole bands shift downward after K deposition, surprisingly, the spectral weight associated with the CB is absent and no Fermi-edge cut-off is observed, in stark contrast to the case of K-deposited BaMg_2_Bi_2_ (Fig. 3c,h,i). This contrast behavior is clearly seen in the EDCs at the Γ point (red curves in Fig. 4c,d). The absence of spectral weight above the VB top cannot be explained in terms of the existence of bulk CB with suppressed intensity, because the photoionization cross-section of Ba 6*s* and Sr 5*s* orbitals that dominantly contribute to the CB^30^ is almost equal to each other at *hν* = 70–80 eV^39^ (also see Supplementary Fig. S3 which shows the absence of the CB-derived intensity in the ARPES data measured along different *k* cuts or at different *hν*’s). It is thus concluded that an intrinsic band gap opens in SrMg_2_Bi_2_. This suggests the occurrence of topological phase transition from the TDS state to the insulating phase upon gradually replacing Ba with Sr.

Now we discuss the origin of observed drastic difference in the near-*E*_F_ band structure between BaMg_2_Bi_2_ and SrMg_2_Bi_2_. According to the calculation^30^, the existence of bulk Dirac-cone band in BaMg_2_Bi_2_ is associated with the protection of band degeneracy along the ΓA line by C_3_ rotational symmetry of the crystal, besides the band inversion of bulk VB and CB with different parities. Taking into account these conditions, one can think of two possibilities to account for the transition to the bulk insulating phase upon replacement of Ba with Sr. One is a transition to the TI phase caused by the C_3_-symmtery breaking, and another is transformation into an ordinary insulator by dissolving the band inversion. The former possibility is ruled out because (i) SrMg_2_Bi_2_ has the same space group as BaMg_2_Bi_2_ so that the C_3_ symmetry is preserved, and (ii) no evidence for the topological Dirac-cone surface state was found within the bulk band gap. One may think that crystal imperfections such as disorders, defects, vacancies, and/or strain may break the C_3_ symmetry. To break the C_3_ symmetry globally and open a large Dirac gap throughout the crystal, a substantial amount of disorders, defects, and/or vacancies must be present in our SrMg_2_Bi_2_ crystals. Local symmetry breaking by a small amount of imperfections unlikely causes a clear gap opening in the ARPES spectrum because ARPES reflects information of photoelectron signal emitted from a wide area over few-tens of micrometers. While such substantial disorders, defects, and/or vacancies may lead to significant spectral broadening in SrMg_2_Bi_2_, our ARPES results indicate that the sharpness of ARPES spectrum is comparable between SrMg_2_Bi_2_ and BaMg_2_Bi_2_ (e.g., compare Fig. 4a and Supplementary Fig. S1b). Therefore, a clear gap opening observed only in SrMg_2_Bi_2_ is unlikely triggered by disorders, defects, and/or vacancies. Also, it is unlikely that accidentally introduced strain in the crystal could globally and reproducibly break the C_3_ symmetry (note that we have confirmed the reproducibility of the gap opening for different crystals). Therefore, the crystal imperfection would not be a main cause of the observed gap in SrMg_2_Bi_2_. Thus, the latter possibility, transformation into an ordinary insulator, is likely the case. This conclusion is reasonable because the replacement of Ba with lighter Sr reduces the strength of SOC and may dissolve the band inversion (Fig. 4e) (note that SrMg_2_Bi_2_ can be either a trivial insulator or a TDS with a small band inversion by our calculations, depending on the approximation used, likely because SrMg_2_Bi_2_ is in the vicinity of the topological phase transition; for details, see Supplementary Fig. S5).

Finally, we compare the TDS characteristics of BaMg_2_Bi_2_ with other well-known TDSs, Na_3_Bi and Cd_3_As_2_^5,7,15–17^. These TDSs share several common features, for example, the Dirac-cone band is protected by the rotational (C_3_ or C_4_) symmetry of crystal and the band inversion is associated with the *s* orbital of CB and *p* orbital of VB. We found that the Fermi velocity of BaMg_2_Bi_2_ (4.2 eV Å) is comparable to that of Na_3_Bi (2.4–2.8 eV Å)^5^ and Cd_3_As_2_ (8.47–9.8 eV Å)^16,17^, implying a potential to achieve a high mobility in BaMg_2_Bi_2_. Also, the SOC of BaMg_2_Bi_2_ is as strong as that of Na_3_Bi and Cd_3_As_2_ because the Dirac-cone band is formed by the orbital of heavy elements, i.e. Bi 6*p* and Ba 6*s*. Regarding the robustness of crystal against the exposure to the air, BaMg_2_Bi_2_ and Cd_3_As_2_ have an advantage over Na_3_Bi, because Na_3_Bi is known to be very unstable in the atmosphere. It is noted that our *X*Mg_2_Bi_2_ crystals were stable for at least 1 week in the atmosphere. The robustness is important for reliable measurements of physical properties as well as applications to practical devices. Most intriguing aspect of BaMg_2_Bi_2_ compared to other TDSs is that chemical substation can be carried out with ease; e.g. Ba atoms can be completely replaced with other elements in BaMg_2_Bi_2_ whereas only partial Cd or As can be replaced with other elements in Cd_3_As_2_^40,41^ and even partial substitution is hard to be done in Na_3_Bi. These characteristics make the *X*Mg_2_Bi_2_ system an excellent platform to investigate the topological phase transition starting from the TDS phase.

In conclusion, we have reported the ARPES results of *X*Mg_2_Bi_2_ (*X* = Sr and Ba). Through the band-structure mapping in the 3D BZ by utilizing energy-tunable SX and VUV photons, we revealed that the electronic structure of BaMg_2_Bi_2_ is characterized by the touching of bulk VB and CB around the Γ point, supporting its TDS nature. On the other hand, a sizable bulk band gap observed in SrMg_2_Bi_2_ suggests its ordinary insulator nature. We concluded that the chemical substitution in *X*Mg_2_Bi_2_ works as an effective means to control the topological phase. The present result paves a pathway toward understanding the interplay among SOC, crystal symmetry, and topological phase transition in TDSs.

## Methods

### Sample preparation

*X*Mg_2_Bi_2_ single crystals were synthesized by the self-flux method. The purity of starting materials is > 99% for Sr and Ba, > 99.9% for Mg, and 99.999% for Bi, respectively. Those materials were loaded in an alumina crucible with the molar ratio of *X*:Mg:Bi = 1:4:6 and the heat treatment was performed in a sealed quartz tube. The growth process is essentially the same as the previous report^32^, including removal of the flux with a centrifuge at a high temperature of ~ 650 °C. A 2θ–θ X-ray diffraction scan confirmed that the growth facet is (0001) plane and the obtained *c*-axis lattice constant is consistent with that of the target phase.

### ARPES experiments

VUV-ARPES measurements with micro-focused synchrotron light were performed with a DA30 electron analyzer at BL28 in Photon Factory and also with a MBS-A1 analyzer at BL5U in UVSOR. We used circularly/linearly polarized light of 36–200 eV. SX-ARPES measurements were performed with a SES2002 analyzer at BL2 in Photon Factory with 230–600 eV photons with horizontal linear polarization. The energy resolutions for VUV- and SX-ARPES measurements were set to be 10–30 meV and 150 meV, respectively. Samples were cleaved in situ along the (0001) plane of the hexagonal crystal in an ultrahigh vacuum of 1 × 10^–10^ Torr, and kept at *T* = 4.5–40 K during the measurements.

### Calculations

First-principles band-structure calculations shown in Figs. 1, 2 and 3 were carried out by using the Quantum Espresso code package^42^ with generalized gradient approximation (GGA)^43^. Ultrasoft pseudopotential was used and the SOC was included in the calculations. The plane-wave cutoff energy and the *k*-point mesh were set to be 70 Ry and 8 × 8 × 8, respectively. We have also performed band-structure calculations by using a projector augmented wave method implemented in Vienna Ab initio Simulation Package (VASP) code^44^ with GGA, local-density approximation (LDA), and Heyd–Scuseria–Ernzerhof (HSE06) hybrid functional, as shown in Supplementary Fig. S5.

## Supplementary Information

Supplementary Information.

## Data Availability

The data that support the findings of this study are available from the corresponding authors upon reasonable request.
