# Supplementary material for: Dirac semimetal phase and switching of band inversion in XMg2Bi2 (X = Ba and Sr)
Source: Sci Rep. 2021 Nov 9;11:21937. doi: 10.1038/s41598-021-01333-z (PMC8578568; doi:10.1038/s41598-021-01333-z)
Supplement: Supplementary file 1 — Supplementary Information. [file 41598_2021_1333_MOESM1_ESM.pdf]

Supplementary Information for "Dirac semimetal phase and switching of band inversion in XMg$_2$Bi$_2$ (X = Ba and Sr)"

Daichi Takane,[1] Yuya Kubota,[1] Kosuke Nakayama,[1,2] Tappei Kawakami,[1] Kunihiko Yamauchi,[3] Seigo Souma,[4,5] Takemi Kato,[1] Katsuaki Sugawara,[1,2,4,5] Shin-ichiro Ideta,[6,7,8] Kiyohisa Tanaka,[6,7] Miho Kitamura,[9] Koji Horiba,[9,10] Hiroshi Kumigashira,[11] Tamio Oguchi,[12,13] Takashi Takahashi,[1,4,5] Kouji Segawa,[14] and Takafumi Sato[1,4,5]

[1]*Department of Physics, Graduate School of Science, Tohoku University, Sendai 980-8578, Japan*

[2]*Precursory Research for Embryonic Science and Technology (PRESTO), Japan Science and Technology Agency (JST), Tokyo, 102-0076, Japan*

[3]*Center for the Promotion of Interdisciplinary Education and Research, Kyoto University, Kyoto, 606-8501, Japan*

[4]*Center for Spintronics Research Network, Tohoku University, Sendai 980-8577, Japan*

[5]*Advanced Institute for Materials Research (WPI-AIMR), Tohoku University, Sendai 980-8577, Japan*

[6]*UVSOR Synchrotron Facility, Institute for Molecular Science, Okazaki 444-8585, Japan*

[7]*School of Physical Sciences, The Graduate University for Advanced Studies (SOKENDAI), Okazaki 444-8585, Japan*

[8]*Hiroshima Synchrotron Radiation Center, Hiroshima University, Higashi-Hiroshima 739-0046, Japan*

[9]*Institute of Materials Structure Science, High Energy Accelerator Research Organization (KEK), Tsukuba, Ibaraki 305-0801, Japan*

[10]*National Institutes for Quantum and Radiological Science and Technology (QST), Sayo, Hyogo 679-5148, Japan*

[11]*Institute of Multidisciplinary Research for Advanced Materials (IMRAM), Tohoku University, Sendai 980-8577, Japan*

[12]*Center for Spintronics Research Network, Osaka University, Toyonaka 560-8531, Japan*

[13]*Institute of Scientific and Industrial Research, Osaka University, Ibaraki, Osaka 567-0047, Japan*

[14]*Department of Physics, Kyoto Sangyo University, Kyoto 603-8555, Japan*

Section 1: Valence-band structure of BaMg$_2$Bi$_2$

Figure S1a displays the ARPES intensity as a function of in-plane wave vector at representative binding-energy (E_B) slices measured at $h\nu = 70$ eV for BaMg$_2$Bi$_2$. One can recognize that the obtained energy contours well follow the periodicity of hexagonal

Brillouin zone, irrespective of E_B's. Also, the symmetry of energy contours appears to match that of the Brillouin zone. This confirms that the cleaving plane is (0001). Figure S1b shows a side-by-side comparison of the band dispersions between ARPES and first-principles band-structure calculations. One can see an overall agreement between the ARPES intensity distribution and the calculated band structure at k_z = 0 (ΓKM) plane (solid curves), in particular, the appearance of two holelike bands approaching/crossing the Fermi level (E_F) around the Γ point. A careful look also reveals that the local maxima

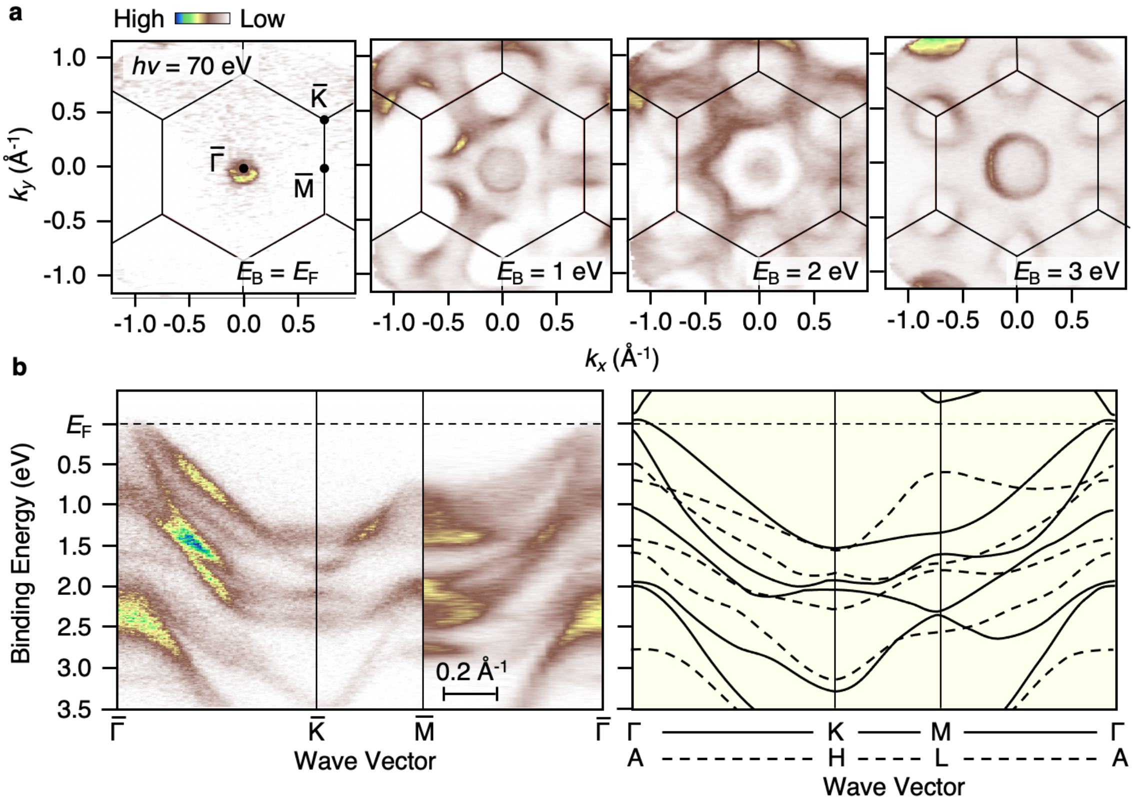

Figure S1: **a** Plots of ARPES intensity as a function of two-dimensional wave vector (k_x and k_y) at representative E_B slices (E_B = 0, 1, 2, and 3 eV) measured at $h\nu$ = 70 eV for $BaMg_2Bi_2$. **b** Comparison of experimental band dispersion measured along high-symmetry lines at $h\nu$ = 70 eV with calculated band dispersions for k_z = 0 (ΓKM; solid curves) and π (AHL; dashed curves) planes.

of the band dispersion seen at the $\bar{\mathrm{M}}$ point around 0.9 eV in the experiment does not well follow the calculated band dispersion for $k_z = 0$. It rather resembles the calculation at $k_z = \pi$ (dashed curve). This signifies the presence of k_z broadening effect.

Section 2: Hall-coefficient measurements of XMg$_2$Bi$_2$

Figure S2a displays the temperature dependence of Hall coefficient R_H for BaMg$_2$Bi$_2$. One can see that R_H increases with decreasing the temperature. The positive R_H over the whole temperature range indicates dominant hole carrier. The hole density p at 50 K is estimated to be 2.4 $\times 10^{18}$ cm^{-3} from $p = 1/eR_H$ (Fig. S2b), in rough agreement with the carrier concentration estimated from the Fermi-surface volume in the ARPES measurements (7 $\times$ 10^{18} cm^{-3}). As shown in Fig. S2c, positive R_H, indicative of dominant hole carrier, is also observed for SrMg$_2$Bi$_2$. The estimated p at 50 K (7.3 $\times 10^{18}$ cm^{-3}) is

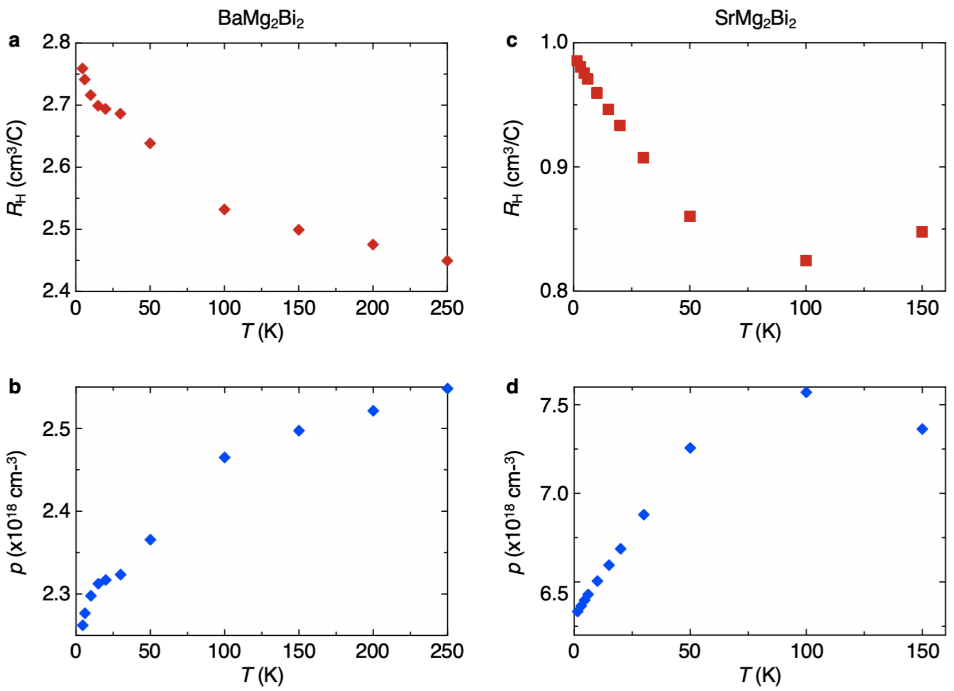

Figure S2: **a**, **b** Temperature dependence of Hall coefficient R_H and carrier density p, respectively for BaMg$_2$Bi$_2$. **c**, **d** Same as **a** and **b**, respectively, but for SrMg$_2$Bi$_2$.

about 3 times larger than that for $BaMg_2Bi_2$. Such an increase in p is reflected in the ARPES data where the hole band of $SrMg_2Bi_2$ crosses E_F at a larger k than that of $BaMg_2Bi_2$ (compare Figs. 3a and 4b).

Section 3: Band gap opening in K-deposited $SrMg_2Bi_2$

Figure S3 displays the ARPES intensity measured along the ΓK and ΓM cuts at $h\nu$ = 80 eV (Figs. S3a and b, respectively) and that along the ΓK cut at $h\nu$ = 110 eV (Fig. S3c) (note that Fig. S3a is reproduced from Fig. 4c). These data commonly show the absence of the spectral weight originating from the conduction band (CB), as confirmed by the absence of the Fermi-edge cut-off in the ARPES spectrum at the Γ point (red curve), supporting the band gap opening in $SrMg_2Bi_2$. It is noted that, besides the CB at the Γ point, that at the M point is also located above E_F at this doping level (see Supplementary Fig. S4).

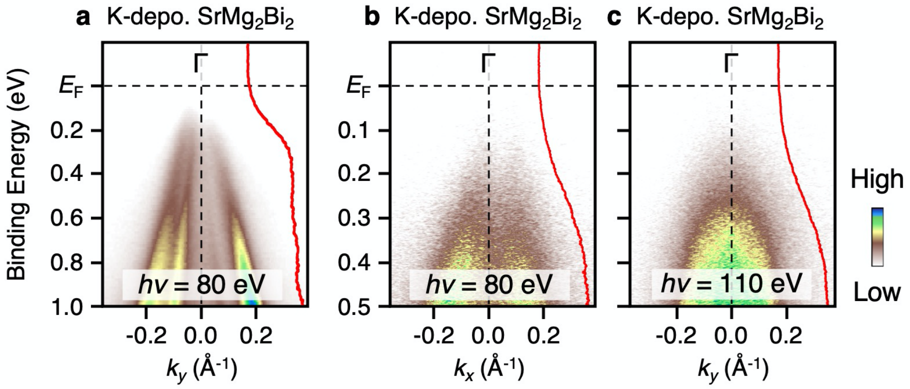

Figure S3: **a** ARPES intensity measured along the ΓK cut at $h\nu$ = 80 eV in K-deposited $SrMg_2Bi_2$. Red curve is the energy distribution curve at the Γ point. **b** Same as **a** but measured along the ΓM cut. **c** Same as **a** but measured at $h\nu$ = 110 eV.

Section 4: The CB at the M point

Our calculations predict that the bottom of the electron-like dispersion at the M point is located at 0.26 eV and 0.31 eV above E_F for pristine $BaMg_2Bi_2$ and $SrMg_2Bi_2$, respectively. Since these values are comparable to the energy shift observed in ARPES measurements for K-deposited samples (0.2-0.3 eV) and the electron-like band is absent in the ARPES data as shown in Fig. S4, the bottom of electron-like band at the M point would be slightly above E_F in the present study.

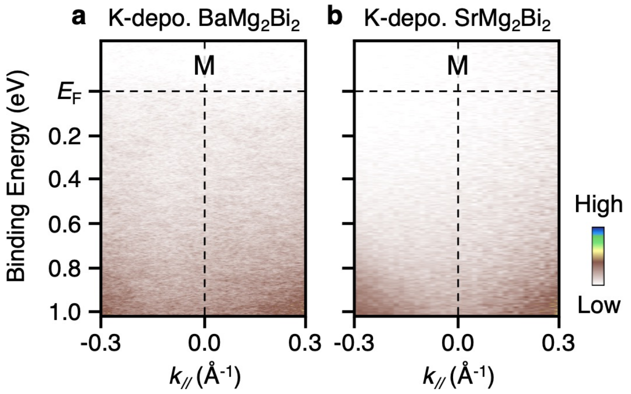

Figure S4: **a**, **b** ARPES intensity measured along a *k* cut crossing the M point in K-deposited $BaMg_2Bi_2$ and K-deposited $SrMg_2Bi_2$, respectively.

Section 5: Band calculations of $SrMg_2Bi_2$

Figure S5 shows a comparison of first-principles band-structure calculations around the Γ point of $SrMg_2Bi_2$, performed by using a projector augmented wave method implemented in VASP code with (a) LDA, (b) GGA, and (c) HSE06 hybrid functional. The calculated bulk bands with LDA and HSE06 show a gap opening between VB and CB (Figs. S5a and c), qualitatively consistent with the preset ARPES result on $SrMg_2Bi_2$, whereas the calculation with GGA shows a TDS state as represented by the presence of a Dirac-band crossing on the ΓA axis (Fig. S5b). These results indicate variation of the

topological property of $SrMg_2Bi_2$ depending on the calculation method, suggesting that $SrMg_2Bi_2$ is in the vicinity of the topological phase transition.

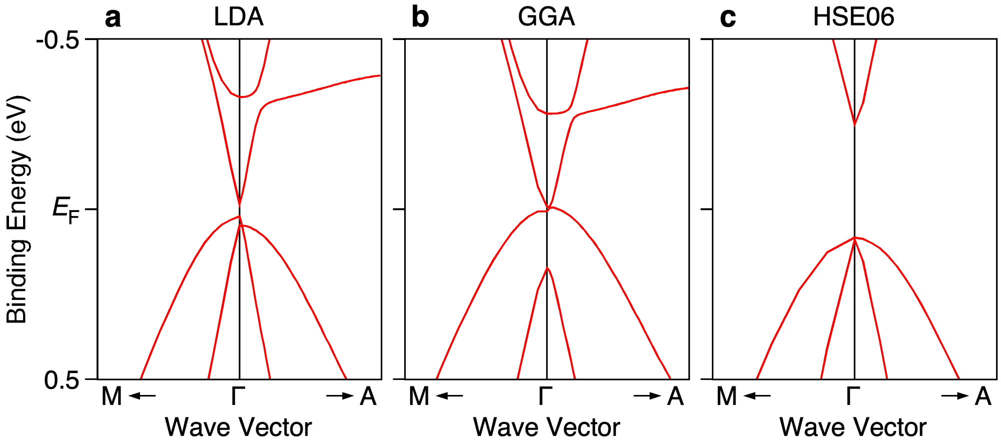

Figure S5: **a-b** Bulk band dispersions along the ΓM and ΓA cuts in $SrMg_2Bi_2$ obtained with LDA, GGA, and HSE06 calculations, respectively. In the calculations, the crystal structure was fully optimized and the SOC was included self-consistently.
